# AM404, paracetamol metabolite, prevents prostaglandin synthesis in activated microglia by inhibiting COX activity

**DOI:** 10.1186/s12974-017-1014-3

**Published:** 2017-12-13

**Authors:** Soraya Wilke Saliba, Ariel R. Marcotegui, Ellen Fortwängler, Johannes Ditrich, Juan Carlos Perazzo, Eduardo Muñoz, Antônio Carlos Pinheiro de Oliveira, Bernd L. Fiebich

**Affiliations:** 1 Department of Psychiatry and Psychotherapy, Laboratory of Translational Psychiatry, Faculty of Medicine, Medical Center – University of Freiburg, Hauptstr. 5, 79104 Freiburg, Germany; 2 Faculty of Biology, University of Freiburg, Freiburg, Germany; 3 Laboratory of Hepatic Encephalopathy and Portal Hypertension, Center of Applied and Experimental Pathology, University of Buenos Aires, Buenos Aires, Argentina; 4 Departamento de Biología Celular, Instituto Maimónides de Investigación Biomédica de Córdoba, Hospital Universitario Reina Sofía, Fisiología e Inmunología, Universidad de Córdoba, Córdoba, Spain; 5 Department of Pharmacology, Universidade Federal de Minas Gerais, Belo Horizonte, MG Brazil

**Keywords:** AM404, Acetaminophen, Microglia, Inflammation, Prostaglandins, Cyclooxygenase

## Abstract

**Background:**

*N*-arachidonoylphenolamine (AM404), a paracetamol metabolite, is a potent agonist of the transient receptor potential vanilloid type 1 (TRPV1) and low-affinity ligand of the cannabinoid receptor type 1 (CB1). There is evidence that AM404 exerts its pharmacological effects in immune cells. However, the effect of AM404 on the production of inflammatory mediators of the arachidonic acid pathway in activated microglia is still not fully elucidated.

**Method:**

In the present study, we investigated the effects of AM404 on the eicosanoid production induced by lipopolysaccharide (LPS) in organotypic hippocampal slices culture (OHSC) and primary microglia cultures using Western blot, immunohistochemistry, and ELISA.

**Results:**

Our results show that AM404 inhibited LPS-mediated prostaglandin E_2_ (PGE_2_) production in OHSC, and LPS-stimulated PGE_2_ release was totally abolished in OHSC if microglial cells were removed. In primary microglia cultures, AM404 led to a significant dose-dependent decrease in the release of PGE_2_, independent of TRPV1 or CB1 receptors. Moreover, AM404 also inhibited the production of PGD_2_ and the formation of reactive oxygen species (8-iso-PGF_2_ alpha) with a reversible reduction of COX-1- and COX-2 activity. Also, it slightly decreased the levels of LPS-induced COX-2 protein, although no effect was observed on LPS-induced mPGES-1 protein synthesis.

**Conclusions:**

This study provides new significant insights about the potential anti-inflammatory role of AM404 and new mechanisms of action of paracetamol on the modulation of prostaglandin production by activated microglia.

**Electronic supplementary material:**

The online version of this article (10.1186/s12974-017-1014-3) contains supplementary material, which is available to authorized users.

## Background

Acetaminophen (N-acetyl-para-aminophenol or paracetamol) was introduced in the market more than a century ago. This compound is one of the most common prescribed and over-the-counter (OTC) drugs in the world, although its mechanism of action is not fully understood. It has been shown that acetaminophen has good analgesic and antipyretic properties, but a weak anti-inflammatory activity, inhibiting the prostaglandin synthesis in the central nervous system (CNS) [1], but not peripherally [2].

Acetaminophen undergoes a deacetylation to *p*-aminophenol not only in the liver but also in the CNS [3]. In the CNS, p-aminophenol is conjugated with arachidonic acid by the fatty acid amide hydrolase (FAAH) to produce *N*-arachidonoylphenolamine (AM404) [3–5]. It has been suggested that AM404 may be responsible for the analgesic mechanism of paracetamol [3, 6, 7].

The pharmacokinetics of AM404 formation in the CNS after acetaminophen administration has been studied by two different groups, Högestätt et al. [3] and Murasamatsu et al. [7]. Högestätt and collaborators have shown after 20 min of intraperitoneal injections of acetaminophen (30, 100, and 300 mg/kg) or p-aminophenol (10, 30, and 100 mg/kg), these compounds were converted to AM404 at the doses of 0.14, 1.6, and 10.3 pmol/g and 3.2, 44, and 667 pmol/g, respectively [3]. Murasamatsu et al. (2016) demonstrated the conversion of acetaminophen in AM404 in rats treated orally with acetaminophen (20 mg/kg), and the peak of AM404 concentration was 150 pg/g at the half-life value of 0.3 h.

AM404 is a potent agonist of the transient receptor potential vanilloid type 1 (TRPV1) [8], a low-affinity ligand of the cannabinoid receptor type 1 (CB1) [3, 9], and an anandamide membrane transporter (AMT) blocker [10, 11]. Furthermore, it has been shown that AM404 induces hypothermia [12, 13] and analgesia in animal models [14–16].

Some studies have demonstrated the effect of AM404 in modulating inflammation and oxidative stress. Its effects on reducing oxidative stress have been associated with the presence of a phenolic group in its structure [17, 18]. AM404 decreased the production of the cytokines interleukin (IL)-1β and IL-6 and increased in circulating tumor necrosis factor (TNF)-α levels in a murine model of inflammation induced by LPS injection [19]. In a rat model of neuropathic pain, AM404 prevented the overproduction of nitric oxide (NO) and TNF-α and increased IL-10 production [14]. Furthermore, this acetaminophen metabolite inhibited the prostaglandin (PG) E_2_ formation and the activity of isolated cyclooxygenase (COX)-1 and COX-2 enzymes ex vitro, and in RAW264.7 macrophages [3]. In human T cells, AM404 is a potent inhibitor of T cell receptor (TCR)-mediated T cell activation and specifically inhibited both IL-2 and TNF-α gene transcription and TNF-α protein synthesis in CD3/CD28-stimulated Jurkat T cells in a FAAH independent way regulating the activation of the transcription factors NF-κB, NFAT, and AP-1 [20]. In an animal model, it has been shown to inhibit both isoforms of prostaglandin endoperoxide synthase/cyclooxygenases (PGHS/COX-1 and -2) [21].

Although there are few studies suggesting that AM404 modulates inflammation, the effects of AM404 on the production of members of the arachidonic acid pathway in activated microglia and brain tissues have not been elucidated in detail, possible explaining some mechanism of actions of acetaminophen in the CNS. We therefore investigated the effects of AM404 on the production of eicosanoids induced by LPS in organotypic hippocampal slice cultures (OHSC) and activated primary microglia.

## Methods

### Reagents

AM404 was obtained from Tocris Biosciences (Ellisville, MO) and dissolved in dimethyl sulfoxide (DMSO - Merck KGaA, Darmstadt, Germany) to get a 50 mM stock solution. Further dilutions in DMSO were prepared immediately before the stimulation*.* AM251 (CB1 antagonist) and capsazepine (TRPV1 antagonist) (Tocris Biosciences) were dissolved in DMSO. LPS from *Salmonella typhimurium* (Sigma-Aldrich, Taufkirchen, Germany) was resuspended in Dulbecco’s Phosphate Buffered Saline (DPBS; Gibco^®^ by Life Technologies, Germany) as 5 mg/mL stock and was used at a final concentration of 10 ng/mL in the microglial culture and 100 ng/mL in OHSC. Solvent concentration in the culture media was maintained at 0.1%.

### Preparation of organotypic hippocampal slice cultures (OHSC)

OHSC of C57Bl/6 wild-type (WT) mice were prepared as previously described [22]. In brief, 2 to 3 days old animals, under sterile conditions, were decapitated; the hippocampi were dissected and placed in a tissue chopper (McIlwain™) for the preparation of 350 μm slices. The slices were transferred to a 0.4 μm culture plate inserts (Millipore, PICM03050). Each insert containing 6 slices were placed in a six-well culture plate containing 1 mL of culture medium [0.5× minimum essential medium (MEM), 25% horse serum, 25% basal medium without glutamate (BME), 2 mM glutamax, and 0.35% glucose]. Then, the slices were incubated at 35 °C in humidified atmosphere with 5% CO_2_ (Heracell 240i, Thermo Scientific). The culture medium was changed after the first day of preparation following every 2 days. After 1 week, the slices were pre-incubated with DMSO 0.1% or AM404 50 μM [23] for 30 min and then stimulated with LPS for 24 h.

### Depletion of microglia from slices culture

For the depletion of the microglia from the slice cultures, 1:10 of liposome-encapsulated clodronate (Lip-CL - Merck Chemicals GmbH, Darmstadt, Germany) solution (0.5 mg/mL) was used. After preparation of OHSC, as described above, the slices were incubated overnight (35 °C in humidified atmosphere with 5% CO_2_ - Heracell 240i, Thermo Scientific) with culture medium containing Lip-CL. Then, slices were rinsed in DPBS at 35 °C and cultured in standard culture medium for another 6 days [22]. This procedure reduces the microglia population to less than 5%, but it does not affect the astrocytes and neuron cells [22, 24].

### Immunohistochemistry

After 1 week of OHSC preparation (wild-type––WT or microglia depleted––Lip-CL), OHSC were washed with DPBS followed by 4% paraformaldehyde (PFA) incubation for 1 h. After fixation, the slices were washed with DPBS and incubated with 5% normal goat serum (NGS - Vector) in DPBS containing 0.3% Triton X-100 (DPBS^+^) for at least 2 h. Subsequently, the slices were incubated overnight with mouse anti-GFAP (1:1000, Cell signaling), anti-rabbit-Iba-1 (1:1000, Wako), and DAPI (1:1000, Sigma) in 1% NGS/DPBS^+^ at 4 °C. Then, slices were incubated with the secondary antibodies for 2 h at room temperature. Rabbit highly cross-adsorbed AlexaFluor 594, and mouse AlexaFluor 488 secondary antibody (Invitrogen, Carlsbad, CA, USA) was used to detect Iba-1 or GFAP, respectively. Slices were imaged in a Zeiss microscope (Zeiss, Oberkochen, Germany).

### Primary rat and mouse microglial cell culture

Primary mixed glial cell cultures were prepared from cerebral cortices of 1-day neonatal Sprague-Dawley rats or C57Bl/6 mice or TPRV1^−/−^ knockout mice, as described previously [25, 26]. Briefly, 7–13 forebrains were removed, homogenized, and filtered through a 70-μm cell strainer (BD biosciences, Heidelberg, Germany), under sterile conditions. After centrifugation (1000 rpm, 10 min), cells were collected and resuspended in Dulbecco’s Modified Eagle’s Medium (DMEM) containing 10% fetal calf serum (Biochrom AG, Berlin, Germany) and antibiotics (40 U/mL penicillin and 40 μg/mL streptomycin, both from PAA Laboratories, Linz, Austria) and cultured (5 × 10^5^ cells/plate) on 10-cm cell culture dishes (Falcon, Heidelberg, Germany) in 10% CO_2_ at 37 °C (Heracell 240i, Thermo Scientific). Floating microglia were harvested every week (2–7 weeks) and re-seeded into 75 cm^2^ culture flask to give pure microglial cultures. On the next day, medium was changed to remove non-adherent cells, and after 1 h, the cells were stimulated for respective experiments.

### Cytotoxicity assay

Cytotoxicity assay was performed using CellTox™ Green Cytotoxicity assay kit (Promega, Mannheim, Germany). Briefly, cells were cultured in 96-well plates at the density of 25 × 10^3^ cells/well in DMEM medium containing 10% fetal calf serum (Biochrom AG, Berlin, Germany) and antibiotics (40 U/mL penicillin and 40 μg/mL streptomycin, both from PAA Laboratories, Linz, Austria). Cells were pre-treated with different concentrations of AM404 (0.1–10 μM) or DMSO 0.1% for 30 min. Thereafter, cells were incubated with or without LPS for the next 24 h. Ethanol (10% end conc., Sigma-Aldrich, Taufkirchen, Germany) was used as positive control to induce the cell death. After incubation (10% CO_2_ at 37 °C - Heracell 240i, Thermo Scientific), 100 μl of CellTox™ Green reagent were added in each well. The plate was mixed for 1 min and incubated for 15 min at room temperature, and the fluorescence was measured at 490 nm_Ex_/530 nm_Em_ using a Modulus™ II Microplate Multimode Reader (Turner BioSystems, USA).

The principle of the assay is to evaluate the alterations in the membrane integrity, using the cyanine dye. The dye binds in the dead-cell DNA and enhanced the fluorescent property, which is excluded from viable cells. The fluorescence intensity values obtained were normalized and presented as the percentage of untreated controls.

### Determination of prostaglandin (PG) D_2_, PGE_2_, and 8-iso-prostaglandin F_2α_ (8-iso-PGF_2α_) release by enzyme-linked immunosorbent assay (ELISA)

Microglial cells were pre-treated for 30 min with different concentrations of AM404 (0.1–10 μM) or DMSO 0.1%. Thereafter, LPS (10 ng/mL) was added for 24 h. Supernatants were harvested, and levels of PGE_2_ (Assay Designs Inc., Ann Arbor, MI, USA; distributed by Biotrend, Cologne, Germany), PGD_2_, and 8-iso-PGF_2α_ (Cayman Chemicals, Ann Arbor, Michigan, USA) were measured by enzyme immunoassay (EIA) in the medium according to the manufacturer’s instructions. Standards from 7.8 to 500 pg/mL, 78 to 10,000 pg/mL, and 3.9 to 500 pg/mL were used for PGE_2_ (detection limit of 3.25 pg/mL), PGD_2_ (detection limit of 200 pg/mL) and 8-iso-PGF_2α_ (detection limit of 2.7 pg/mL), respectively. Data was normalized to LPS and presented as percentage of change in PGs levels of at least three independent experiments.

### Western blot analysis

For COX-2 and mPGES-1 immunoblotting, microglial cells were left untreated or treated with LPS (10 ng/mL) in the presence or absence of AM404 (0.1–10 μM) for 24 h. Cells were washed with cold phosphate buffered saline (PBS) and added lysis buffer (42 mM Tris-HCl, 1.3% sodium dodecyl sulfate, 6.5% glycerin, 100 μM sodium orthovanadate, and 2% phosphatase and protease inhibitors). Protein estimation was measured using the bicinchoninic acid method (BCA protein determination kit from Pierce, distributed by KFC Chemikalien, Munich, Germany) according to manufacturer’s instructions and bovine serum albumin (BSA, Sigma) used as a standard. For Western blotting, 20 μg of total protein from each sample were subjected to sodium dodecyl sulfate–polyacrylamide gel electrophoresis (SDS-PAGE) under reducing conditions. Then, proteins were transferred onto a polyvinylidene fluoride (PVDF) membrane (Merck Millipore, Darmstadt, Germany) by semi-dry blotting. The membrane was blocked for 1 h at room temperature using Rotiblock (Roth, Karlsruhe, Germany) and incubated with the primary antibody overnight. Primary antibodies were goat anti-COX-2 (M19, 1:500, Santa Cruz Biotechnology, CA, USA), rabbit anti-mPGES-1 (1:200, Cayman Chemical Co., Ann Arbor, MI, USA), and rabbit anti-actin IgG (1:5000, Sigma, Saint Louis, MO, USA). AB-coupled proteins were detected with horseradish peroxidase-coupled rabbit anti-goat IgG (Santa Cruz, 1:100,000 dilution) or goat anti-rabbit IgG (Amersham, 1:25,000 dilution) using enhanced chemiluminescence Western blotting substrate (Biozym Scientific GmbH, Rockford, USA). The quantification of the Western blots was performed using ImageJ. All Western blot experiments were carried out at least three times.

### Cyclooxygenase activity assay

To evaluate the effect of AM404 on COX enzymatic activity, an arachidonic acid assay was performed as previously described [27]. There are currently two well identified cyclooxygenase isoforms, named COX-1 and COX-2. The first one is constitutively expressed in almost every cell and COX-2, although can also be constitutive in some cells, is induced by cytokines and endotoxins [28, 29]. Under unstimulated conditions, primary microglial cells only express the COX-1 isoform [30]. Briefly, to measure COX-1 activity, primary rat microglial cells were plated in 24-well cell culture plates, and after 24 h, the medium was removed and replaced with serum-free medium. AM404 (0.1–10 µM) or selective COX-1 inhibitors [acetylsalicylic acid (ASA, 50–100 μM), irreversible inhibitor, or SC560 (0.1–1 μM) reversible inhibitor] were added, and left for 15 min. After that, 15 µM of arachidonic acid were supplemented for another 15 min. Supernatants were then collected and used for the determination of PGE_2_.

We also investigated the effects of AM404 on microglial total COX (COX-1 + COX-2) enzymatic activity, which mainly determined COX-2 mediated PGE_2_ production. Since we also have a slight contribution of COX-1 activity in this assay, we titled as COX-1 + COX-2 activity.

The COX-1 + COX-2 activity assay was conducted exactly as mentioned before with pre-incubation with LPS (10 ng/mL) for 24 h to induce COX-2 synthesis and using diclofenac sodium (preferential COX-2 inhibitor, 1–10 μM).

Furthermore, to understand if the effects of AM404 in COX-1 and COX-1+COX-2 activities produce a reversible or irreversible inhibition, the assays were evaluated with some modifications. For COX-1 activity, the drugs were incubated for 60 min, after the wells were washed three times with PBS 37 °C and incubated for 3 h with serum-free medium. Then, medium was changed and added 15 µM arachidonic acid for 15 min. For total COX activity, primary rat microglial cells were first pre-incubated with LPS (10 ng/mL) for 24 h and then follow the same procedure describe for COX-1. Finally, the supernatants were collected for determination of PGE_2_.

### Data analysis

Results were converted into percentage values of LPS and presented as mean ± SEM. Data was analyzed using one-way analysis of variance (ANOVA) followed by Newman-Keuls post-test. The level of statistical significance was set as *p < 0.05, **p < 0.01, and ***p < 0.001. Graph Pad Prism (Graph Pad Software, San Diego, CA) was used for performing all statistical analysis.

## Results

### AM404 inhibited LPS-induced PGE_2_ release by microglia in OHSC

**Fig. 1:**
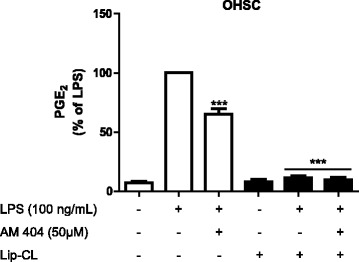
AM404 reduces LPS-induced PGE_2_ release in OHSC, and this effect is mediated by microglial cells. In wild-type OHSC (OHSC WT, *white bars*) and microglia-depleted OHSC (OHSC LIP-CL, *black bars*), AM404 was added 30 min before stimulating with LPS and the amount of PGE_2_ in the culture medium was determined after 24 h using an enzyme immunoassay. Each column and error bar represents the mean ± SEM of four OHSC/group (six slices/well). *p < 0.05, **p < 0.01, and ***p < 0.001 with respect to LPS (one-way ANOVA followed by the Newman-Keuls *post-*test)

We first investigated whether AM404 reduces LPS-induced PGE_2_ release in organotypic hippocampal slices cultures (OHSCs). As shown in Fig. 1, the production of PGE_2_ after 24 h was increased after LPS stimulation and AM404 50 μM prevented the synthesis of PGE_2_ in OHSC (Fig. 1). Furthermore, to determine the involvement of microglia in LPS-mediated PGE_2_ levels in OHSC, we depleted these cells by using a liposome-encapsulated clodronate solution (Lip-CL) as previously demonstrated [22]. Incubation of the cells with Lip-CL reduced the microglia population to less than 5%, but not affecting astrocytes and neurons [22, 24] (Additional file 1). After the depletion of microglia in OHSC, a potent reduction on PGE_2_ release was observed after stimulation with LPS (Fig. 1, black bars), demonstrating that the PGE_2_ inducing effect of LPS directly depends of microglial cells.

### AM404 inhibited LPS-induced PGE_2_ release in primary microglial cell cultures

**Fig. 2:**
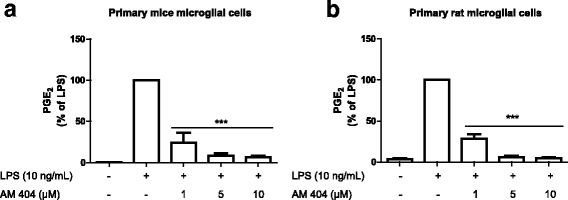
AM404 concentration dependently reduces LPS-induced PGD_2_ **(a)** and 8-isoprostane **(b)** release after LPS stimulation in primary rat microglial cells. AM404 was added 30 min before stimulating with LPS, and the amount of PGD_2_ **(a)** and 8-iso-PGF_2α_ **(b)** in the culture medium were determined after 24 h using an enzyme immunoassay. Each column and error bar represents the mean ± SEM of five new cultures/group. *p < 0.05, **p < 0.01, and ***p < 0.001 with respect to LPS (one-way ANOVA followed by the Newman-Keuls post-test)

To study the effects of AM404 in microglial cells, we investigated whether AM404 reduces LPS-induced PGE_2_ release in primary mice or rat microglial cell cultures. As shown in Fig. 2, activation of microglial cells with LPS increased the production of PGE_2_ after 24 h. Pre-treatment with AM404 (1–10 μM) prevented the increase of PGE_2_ levels in a concentration-dependent manner in both cultures (Fig. 2a–b). Since the effect on cultures from different species was similar, the following experiments were performed in primary microglial cells cultures from rats.

To exclude that the observed inhibitory effects of AM404 is due to reduced cell viability, a cytotoxicity assay was performed. As shown in Additional file 2, AM404 did not show any significant cytotoxicity in rat microglial cells at the concentrations used.

### The effect of AM404 on reduction of LPS-induced PGE_2_ release is independent of CB1 or TRPV1 receptors

**Fig. 3:**
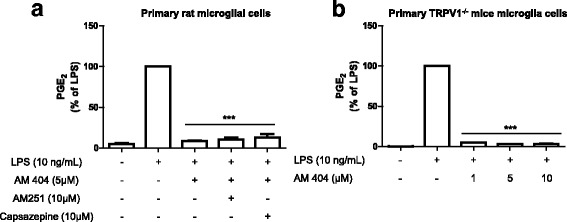
Reduction of LPS-induced PGE_2_ release by AM404 does not involve the CB1 or TRPV1 receptors. Antagonists of the CB1 (AM251) or TRPV1 receptor (Capsazepine) were used before AM404 and LPS treatment in primary rat microglial cells (**a**). Effects of AM404 in LPS-treated primary TRPV1^−/−^ knockout mice microglia cells (**b**). ***p < 0.001 with respect to LPS (one-way ANOVA followed by the Newman-Keuls *post-*test, three new cultures/group)

AM404 is described as an agonist of TRPV1 [8] and CB1 receptors [3, 9]. We therefore aimed to investigate whether AM404 reduced PGE_2_ release (Fig. **2**) is mediated by one or both receptors. To this end, AM251 (CB1 antagonist) or capsazepine (TRPV antagonist) were added 30 min before AM404 and LPS treatment. As shown in Fig. **3**a, the antagonism of CB1 with AM251 (10 μM, −30 min) or TRPV with capsazepine (10 μM, −30 min) did not affect the inhibitory effects of AM404 on PGE_2_ levels. Moreover, AM404 (1, 5 or 10 μM) prevented the increase in PGE_2_ induced by LPS in primary microglia from TRPV1^−/−^ knockout mice underlying the non-involvement of TRPV1 (Fig. **3**b).

### AM404 reduced LPS-induced PGD_2_ and 8-isoprostane release in primary rat microglial cell cultures

**Fig. 4:**
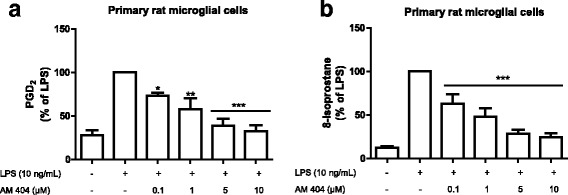
AM404 concentration dependently reduces LPS-induced PGD_2_ (**a**) and 8-isoprostane (**b**) release after LPS stimulation in primary rat microglial cells. AM404 was added 30 min before stimulating with LPS, and the amount of PGD_2_ (**a**) and 8-iso-PGF_2α_ (**b**) in the culture medium were determined after 24 h using an enzyme immunoassay. Each column and error bar represents the mean ± SEM of five new cultures/group. *p < 0.05, **p < 0.01, and ***p < 0.001 with respect to LPS (one-way ANOVA followed by the Newman-Keuls *post-*test)

Subsequently, we tested the effect of AM404 pre-treatment on LPS-induced PGD_2_ release in primary rat microglia. As observed in Fig**.** 4a, the pre-treatment with AM404 (0.1–10 μM) statistically prevented the PGD_2_ release in a concentration-dependent manner.

Next, we evaluated the effect of AM404 on the formation of reactive oxygen species in LPS-activated microglia. 8-iso-PGF_2α_ is produced by the non-enzymatic peroxidation of arachidonic acid in membrane phospholipids and is considered a reliable and highly sensitive marker to assess oxidative stress [31]. As shown in Fig. 4b, stimulation of microglial cells with LPS increased 8-iso-PGF_2α_ levels, which were potently and dose-dependently prevented by pre-treatment with AM404 (0.1–10 μM).

### AM404 reduced COX-2 but not mPGES-1 protein levels in primary rat microglial cell culture

**Fig. 5:**
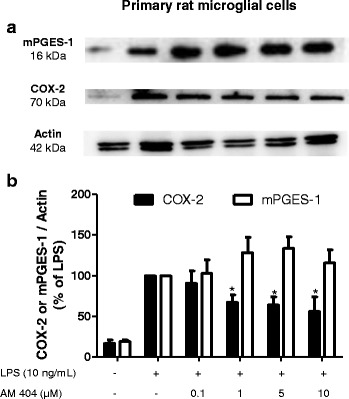
Effects of AM404 on LPS-induced protein levels of COX-2 or mPGES-1 in primary rat microglia cells. **a** Western blot analysis of protein levels of COX-2 or mPGES-1 in LPS-stimulated rat microglia. **b** Quantitative densitometric analysis of COX-2 or mPGES-1 protein levels normalized to β-actin loading control (*n* = 6). *p < 0.01 with respect to LPS (one-way ANOVA followed by the Newman-Keuls *post-*test)

One of the mechanism by which AM404 might reduce prostaglandin levels is the reduction of the expression and synthesis of the enzymes responsible for their synthesis. Thus, we studied whether AM404 affected the synthesis of COX-2 and mPGES-1 in LPS-stimulated rat primary microglial culture. Our data showed that after 24 h of LPS stimulation, AM404 (0.1–10 μM) weakly but significantly reduced the protein levels of LPS-induced COX-2, but it did not affect the synthesis of mPGES-1 increased by LPS (Fig. 5).

### AM404 decreased COX activity in primary rat microglial cell culture

**Fig. 6:**
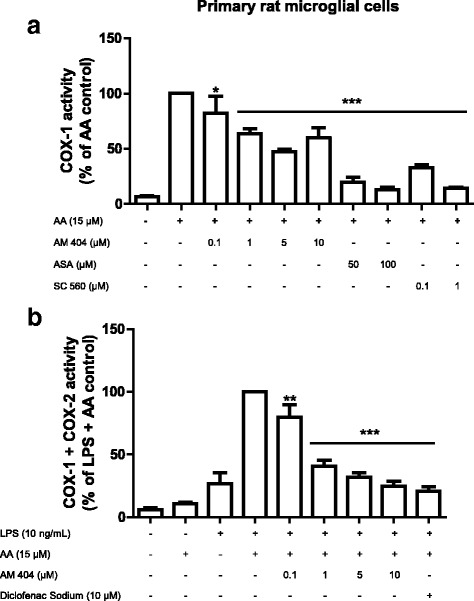
Cyclooxygenase enzymatic activity in primary rat microglia cells is decreased by AM 404. For COX-1 activity assay (**a**), cells were treated with different concentrations of AM 404 (0.1, 1, 5, or 10 μM) for 15 min before the addition of 15 μM of arachidonic acid. PGE_2_ in the supernatants was measured after additional 15 min. For COX-2 (**b**), cells were stimulated for 24 h with LPS (10 ng/mL) and then treated with different concentrations of AM 404 for 15 min. After this incubation time, 15 μM of arachidonic acid was added and PGE_2_ in the supernatants was measured as described in “Methods.” Data is expressed as mean ± SEM of at least three new cultures/group. *p < 0.05, **p < 0.01, and ***p < 0.001 with respect to control (one-way ANOVA followed by the Newman-Keuls *post-*test)

Since AM404 only weakly affected the protein levels of COX-2, we decided to evaluate its effect on COX enzyme activity. First, we evaluated the effect on COX-1 activity and as shown in Fig. 6a, AM404 partially inhibited COX-1 activity (approximately 50%) at the same concentrations that abolished PGE_2_ levels. As expected, both selected inhibitors of COX-1, SC-560, and ASA, potently reduced COX-1 activity.

As demonstrated in Fig. 6b, treatment with AM404 potently inhibited COX-2 activity in a concentration-dependent manner and in a similar profile as the inhibition of LPS-induced PGE_2_ in Fig. 2b.

### AM404 reversibly decreased COX activity in primary rat microglial cell culture

**Fig. 7:**
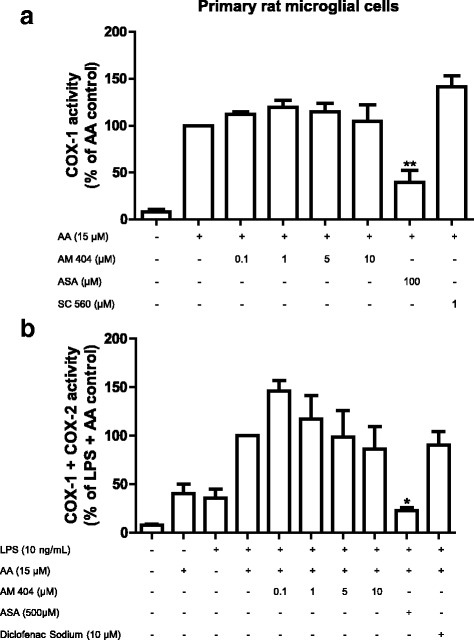
Reversibility of the AM404 mediated inhibition of COX-1 (**a**) and COX-2 (**b**) activity in primary rat microglia cells. **a** The cells were treated with different concentrations of AM 404 (0.1, 1, 5, or 10 μM) for 60 min. For COX-2 (**b**), cells were stimulated for 24 h with LPS (10 ng/mL) and then treated with different concentrations of AM 404 for 60 min. After this incubation time, **a** and **b** were washed three times with DPBS 37 °C and incubated for 3 h with serum-free medium. Fifteen micromolar of arachidonic acid was added, and PGE_2_ in the supernatants was measured as described in “Methods.” Data are expressed as mean ± SEM of at least four new cultures/group). *p < 0.05 and **p < 0.01 with respect to control (one-way ANOVA followed by the Newman-Keuls *post-*test)

To evaluate if the inhibition of COX activity is a reversible or irreversible effect, we removed the AM404 by washing the cultures several times as described in “Methods.” As shown in Fig. 7a, partial inhibition of COX-1 activity by AM404 was abolished after this procedure, and the same effect was observed by SC-560, a reversible inhibitor of COX-1. In contrast, since ASA is an irreversible inhibitor COX-1, the inhibition of the enzyme activity induced by this drug was persistent (Fig. 7a). The same reversible effect of AM404 was observed on the inhibition of COX-2 activity, but in contrast, a high concentration of ASA persistently inhibited the enzyme activity (Fig. 7b).

## Discussion

In the present study, we demonstrated that the acetaminophen metabolite, AM404, reduces inflammatory mediators of the arachidonic acid cascade in OHSC and primary microglial cells. We show here that AM404 decreased the release of PGD_2_, PGE_2_, and 8-iso-PGF_2α_, by a mechanism independent of TRPV1 or CB1 receptors, but by reversibly inhibiting COX-1 and COX-2 activity, as well as by slightly reducing the expression levels of COX-2 protein in microglia.

Various studies have been performed to elucidate the mechanism of action of acetaminophen since it is one of the most prescribed and consumed drugs in the world for mild to moderate pain relieve and fever reduction, and its intracellular pathways are not fully understood. Acetaminophen produces a weak inhibition of COX, and it has been proposed that acetaminophen acts on COX-3, a brain-specific isoform of COX and slice variant of constitutive enzyme COX-1 [32, 33]. Indeed, many studies have demonstrated its inhibitory effect on PGE_2_ production in the brain [1] and in primary microglial culture [2, 34]. PGE_2_ is an important subproduct of the enzymatic action of COX and a mediator of fever where its increase occurs in response to systemic pyrogen agents produced by infectious pathogens [35–38].

Högestätt et al. [3] and Murasamatsu et al. [7] have demonstrated that milligrams of paracetamol are converted in picograms of AM404 into the CNS of rodents, which could induce different pharmacological effects, including the effects on COX expression and activity. However, the correlation between in vitro and in vivo studies is complex due to different pharmacokinetic and micro-environment parameters to be considered. In the meantime, it has been suggested that AM404 is the active metabolite of acetaminophen and is responsible for its analgesic activity [3, 6, 7]. The involvement of AM404 in analgesia has been well established in animal models of nociceptive and neuropathic pain [14–16, 39]. In addition, the effect of AM404 in modulating peripheral inflammation [14, 19, 21] and in immune cell culture [3, 20] has been described. However, the direct effect of AM404 in neuroinflammation, especially in microglia, is still poorly understood.

OHSC is a well-established brain tissue model maintaining the main architecture of the cells, and its complex system provides a good tool to understand cellular and molecular processes of the brain in vitro [40, 41]. Our results in OHSC demonstrated that the AM404 inhibited the release of PGE_2_ induced by LPS stimulation, and by depleting the microglial cells from OHSC, the levels of PGE_2_ after stimulation with LPS were completely abolished. As known, microglial cells are the resident macrophages of the CNS and the most important source of PGE_2_ in neuroinflammation. It has been demonstrated before that in primary microglial cells stimulated with LPS, PGE_2_ and COX-2 expression are strongly increased [42–44].

Thus, to elucidate the mechanisms by which AM404 affects these cells, we continued our further experiments with primary microglial cells (mouse and rat) treated with LPS. Studies in primary cell cultures allowed us to better understanding of the mechanism of pure microglia as a result of distinct stimulations. It has been reported that the process of microglia isolation may lead to their activation [45, 46]. However, we do not observe an activation status in respect of inflammatory parameters. After LPS stimulation, we observe comparable data in cultivated microglia mono-cultures and in organotypic slice culture models, where LPS-induced PGE_2_ release, and therefore believe that our results are robust in both models, although the morphological and biochemical status of microglia may depend on the cells that are also present in the cultured [46–50]. The pre-treatment with AM404 potently prevented the increase on PGE_2_ release stimulated by LPS from both species. This effect of AM404 on downregulating PGE_2_ release provides us an important contribution to explain the mechanism by which paracetamol might control fever. The involvement of AM404 in inhibition of LPS-induced PGE_2_ formation in RAW264.7 macrophages was also described by Högestätt and colleagues [3].

AM404 modulates the endocannabinoid system by acting via TRPV1 and CB1 receptors, and it increased the viability of anandamide in the medium by blocking AMT [10, 11]. The involvement of cannabinoids in inflammation has been shown in different models and demonstrated some evidences for anti-inflammatory effects [51, 52]. Furthermore, many studies have described the participation of cannabinoids on immune modulation [53–56]. Immune cells express the cannabinoid receptors type 1 and type 2, albeit CB2 appears to be the predominant isoform [57, 58]. In microglia, the expression and the amount of CB receptors may change depending on the type of neuropathology. However, it has been reported that rat and mouse primary microglia cultures express both CB1 and CB2 receptor mRNA and protein [52]. Moreover, microglia can also express TRPV1 that mediate some endocannabinoid actions [59, 60]. However, the involvement of TRPV1 and CB1 receptors in AM404 effects is still not completely elucidated since the present data are controversial. Roche et al. [19] have shown that the effect of AM404 on the increase of circulating TNF-α after LPS injection was blocked by SB366791 (TRPV1 antagonist) and AM251 (CB1 antagonist) and the decrease on IL-1ß was attenuated only by AM251, but neither of these antagonists altered the effect of AM404 on the decrease of IL-6. In addition, in a model of cerebral ischemia in gerbils, AM404 reduced neuronal damage, and this effect was reversed by AM251, but not by capsazepine [61]. In the present study, we evaluated the involvement of TRPV1 and CB1 receptors in the modulation of PGE_2_ release by AM404, and our findings showed that the mechanisms underlying the anti-inflammatory effect of AM404 in CNS cells did not involve TRPV1 and CB1 receptors, additionally supported by using TRPV1 knockout mice.

Free radicals also contribute to the inflammatory process by metabolizing arachidonic acid to isoprostanes. The involvement of AM404 on the decrease of oxidative stress has been proposed by the presence of a phenolic group in its structure [17, 18], and Costa et al. [14] proved in a model of neuropathic pain that AM404 prevented the overproduction of nitic oxide (NO). In addition, García-Arencibia and collaborators (2007) have suggested the antioxidant effect of AM404 as one of the neuroprotective mechanism of AM404 in a Parkinson’s disease [62] model. In accordance with the literature, we showed that AM404 prevented the formation of reactive oxygen species in primary microglial cells.

In order to understand the mechanism by which prostaglandins are reduced by AM404, we tested the hypothesis that AM404 inhibits COX activity as described in a monocyte cell line stimulated with LPS (1 μg/mL) [3]. Indeed, we demonstrated that AM404 inhibited COX-1 and COX-2 activities. Furthermore, the inhibition of COX seems to be reversible. Additionally, this compound slightly reduced COX-2 protein levels induced by LPS in microglia. Thus, different mechanisms may participate in the reduction of prostaglandin levels mediated by AM404.

## Conclusions

In summary, we provided evidence that AM404 interferes in several steps of the synthesis of prostaglandins in LPS-activated microglia. This study provides new significant insights on the potential anti-inflammatory activity of AM404 and new mechanisms in respect of the central action of acetaminophen in the modulation of prostaglandin production by microglia.

## Additional files

Additional file 1: Representative images of the immunolabeled OHSC with Iba-1 (red), GFAP (green), and DAPI (blue) in 200 μm. **(A-E)** Wild-type OHSC (OHSC WT) and **(F-I)** microglia-depleted OHSC (OHSC LIP-CL). (PDF 228 kb)

Additional file 2: Effects of AM404 on cell viability in primary rat microglia cells. AM404 was added 30 min before stimulating the cells with LPS for 24 h, and cell death was measured by the intensity of the fluorescence emission in the culture using the CellTox™ Green Cytotoxicity assay kit. Each column and error bar represents the percentage of fluorescence (100% for just microglia cells). ***p < 0.001 with respect to just microglia cells (One-way ANOVA followed by the Newman-Keuls *post-*test). (PDF 16 kb)

## Abbreviations

8-iso-PGF_2α_: 8-Iso-prostaglandin F_2α_
AA: Arachidonic acid
AB: Antibody
AM404: *N*-arachidonoylphenolamine
AMT: Anandamide membrane transporter
AP-1: Activator protein 1
ASA: Acetylsalicylic acid
BCA: Bicinchoninic acid method
BME: Basal medium without glutamate
CB1: Cannabinoid receptor type 1
CNS: Central nervous system
COX: Cyclooxygenase
DAPI: 4′,6-Diamidino-2-phenylindole
DMEM: Dulbecco’s Modified Eagle’s Medium
DMSO: Dimethyl sulfoxide
DPBS: Dulbecco’s Phosphate Buffered Saline
ETOH: Ethanol
FAAH: Fatty acid amide hydrolase
GFAP: Glial fibrillary acidic protein
Iba-1: Ionized calcium-binding adapter molecule 1
IL: Interleukin
Lip-CL: Liposome-encapsulated clodronate
LPS: Lipopolysaccharide
MEM: Minimum essential medium
mPGES: Microsomal prostaglandin E synthase
NFAT: Nuclear factor of activated T cells
NF-kappaB: Nuclear factor kappa-light-chain-enhancer of activated B cells
NGS: Normal goat serum
OHSC: Organotypic hippocampal slices culture
OTC: Over-the-counter
PFA: Paraformaldehyde
PG: Prostaglandin
PGHS: Prostaglandin endoperoxide synthase
PVDF: Polyvinylidene fluoride
SDS-PAGE: Sodium dodecyl sulfate–polyacrylamide gel electrophoresis
TCR: T cell receptor
TLR4: Toll-like receptor 4
TNF: Tumor necrosis factor
TRPV1: Transient receptor potential vanilloid type 1
WT: Wild-type
