# Correction to: AM404, paracetamol metabolite, prevents prostaglandin synthesis in activated microglia by inhibiting COX activity

**DOI:** 10.1186/s12974-018-1072-1

**Published:** 2018-02-06

**Authors:** Soraya Wilke Saliba, Ariel R. Marcotegui, Ellen Fortwängler, Johannes Ditrich, Juan Carlos Perazzo, Eduardo Muñoz, Antônio Carlos Pinheiro de Oliveira, Bernd L. Fiebich

**Affiliations:** 1 Department of Psychiatry and Psychotherapy, Laboratory of Translational Psychiatry, Faculty of Medicine, Medical Center, University of Freiburg, Hauptstr. 5, 79104 Freiburg, Germany; 2 Faculty of Biology, University of Freiburg, Freiburg, Germany; 3 Laboratory of Hepatic Encephalopathy and Portal Hypertension, Center of Applied and Experimental Pathology, University of Buenos Aires, Buenos Aires, Argentina; 4 Departamento de Biología Celular, Instituto Maimónides de Investigación Biomédica de Córdoba, Hospital Universitario Reina Sofía, Fisiología e Inmunología, Universidad de Córdoba, Córdoba, Spain; 5 Department of Pharmacology, Universidade Federal de Minas Gerais, Belo Horizonte, MG Brazil

## Correction

After publication of the article [1], it has been brought to our attention that the caption for Figure 2 has been mistakenly replaced with a reproduction of the Figure 4 caption. Currently the caption for Figure 2 reads “Fig. 2 AM404 concentration dependently reduces LPS-induced PGD2 (a) and 8-isoprostane (b) release after LPS stimulation in primary rat microglial cells. AM404 was added 30 min before stimulating with LPS, and the amount of PGD2 (a) and 8-iso-PGF2α (b) in the culture medium were determined after 24 h using an enzyme immunoassay. Each column and error bar represents the mean ± SEM of five new cultures/group. *p < 0.05, **p < 0.01, and ***p < 0.001 with respect to LPS (one-way ANOVA followed by the Newman-Keuls post-test)” when it in fact read “Fig. 2: AM404 reduces LPS-induced PGE2 release in primary mice (a) and rat (b) microglial cells. AM404 was added 30 min before stimulating microglia with LPS and the amount of PGE2 in the culture medium was determined after 24 h using an enzyme immunoassay. Each column and error bar represent the mean ± S.E.M. of 5 new cultures / group. ***p < 0.001 with respect to LPS (One-way ANOVA followed by the Newman-Keuls post-test).” This has been corrected in the revised version of the article.
