# Supplementary figures and images for: AM404, paracetamol metabolite, prevents prostaglandin synthesis in activated microglia by inhibiting COX activity
Source: J Neuroinflammation. 2017 Dec 13;14:246. doi: 10.1186/s12974-017-1014-3 (PMC5729401; doi:10.1186/s12974-017-1014-3)

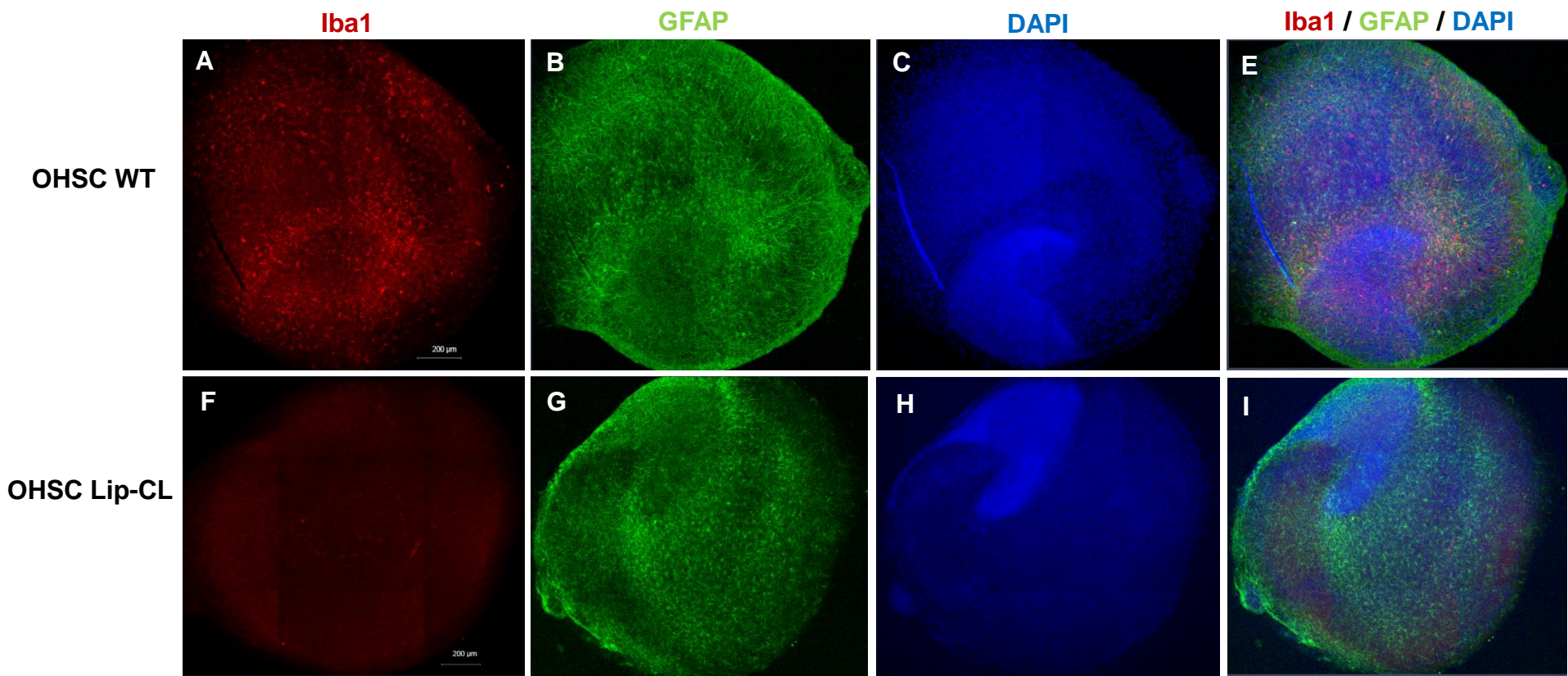

Supplement: Supplementary file 1 — Representative images of the immunolabeled OHSC with Iba-1 (red), GFAP (green), and DAPI (blue) in 200 μm. (A-E) Wild-type OHSC (OHSC WT) and (F-I) microglia-depleted OHSC (OHSC LIP-CL). (PDF 228 kb) [file 12974_2017_1014_MOESM1_ESM.pdf]

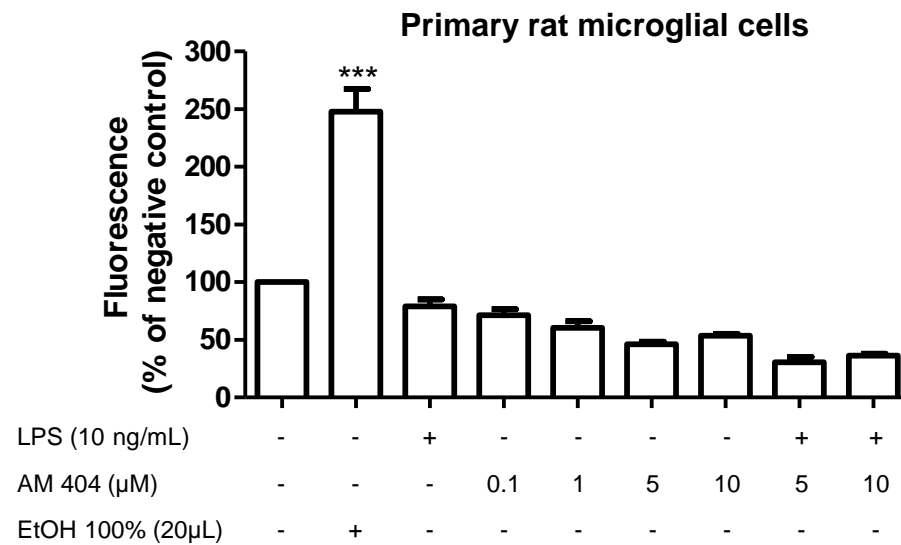

Supplement: Supplementary file 2 — Effects of AM404 on cell viability in primary rat microglia cells. AM404 was added 30 min before stimulating the cells with LPS for 24 h, and cell death was measured by the intensity of the fluorescence emission in the culture using the CellTox™ Green Cytotoxicity assay kit. Each column and error bar represents the percentage of fluorescence (100% for just microglia cells). ***p < 0.001 with respect to just microglia cells (One-way ANOVA followed by the Newman-Keuls post-test). (PDF 16 kb) [file 12974_2017_1014_MOESM2_ESM.pdf]
